# Combatting pain after orthopedic/trauma surgery- perioperative oral extended-release tapentadol vs. extended-release oxycodone/naloxone

**DOI:** 10.1186/s12871-017-0383-6

**Published:** 2017-07-11

**Authors:** Gertrud Haeseler, Dirk Schaefers, Natalie Prison, Jörg Ahrens, Xiaofei Liu, Annika Karch

**Affiliations:** 1 Department of Anesthesia, Katholisches Klinikum Ruhrgebiet Nord (KKRN) GmbH, Hervester Str. 57, D-45768 Marl, Germany; 2 Department of orthopedic and trauma surgery, KKRN, Hervester Str. 57, D-45768 Marl, Germany; 3 Department of anesthesia, KKRN, Hervester Str. 57, D-45768 Marl, Germany; 4 Department of Anesthesia, Hannover Medical School, Carl-Neuberg-Str. 1, D-30625 Hannover, Germany; 5 Institute for Biostatistics, Hannover Medical School, Carl-Neuberg-Str. 1, D-30652 Hannover, Germany

**Keywords:** Post-operative opioid analgesia, Trauma surgery, Oral tapentadol, Oral oxycodone

## Abstract

**Background:**

High post-operative pain scores after “minor” orthopedic/trauma surgery are in part attributed to inadequate prescription of opioid analgesics. Novel concepts aiming to achieve sufficient analgesia while minimizing opioid-related side effects by avoiding fluctuating plasma levels are based on perioperative oral administration of extended-release opioids beginning with the first dose pre-operatively. This is the first study to evaluate analgesic efficacy and side effect rates of extended-release tapentadol compared to oxycodone/naloxone following orthopedic/trauma surgery.

**Methods:**

This randomized, observer-blinded, active-controlled prospective clinical trial had 2 co-primary endpoints: (1) Analgesic efficacy: Mean pain level on a numeric rating scale (NRS) from 0 to 10 during exercise over 5 days. (2) Safety: Side effect sum score of the following events: Nausea, vomiting, constipation, sedation, vertigo, somnolence. The study was powered to detect superiority of tapentadol for at least one endpoint pending statistical proof of non-inferiority for both endpoints in a first step.

**Results:**

Two hundred sixty-six trauma patients were randomized to receive either tapentadol (*n* = 133) or oxycodone/naloxone (*n* = 133). Analgesic efficacy: Mean (±SD) daily pain levels in the first five post-operative days were 2.8 ± 1.3 in both groups. Mean maximum pain intensity during exercise in the first 24 h after surgery was 3.8 ± 1.9 (tapentadol) and 3.8 ± 2.1 (oxycodone/naloxone). Statistically tapentadol was non-inferior but not superior to oxycodone/naloxone. Safety: Vomiting on day 1 occurred in 11%, constipation in 35% of the tapentadol patients and in 16% and 30% of the oxycodone/naloxone patients (*p* = 0.60 and 0.33), respectively. The incidence of sedation/ vertigo was <10%, that of somnolence <2% in both groups (*p* > 0.3, respectively). The sum score of side effect events was 51% in the tapentadol vs. 49% in the oxycodone/naloxone group; risk difference 3% [95% CI, −8 to 14%]; *p* = 0.6). Non-inferiority of tapentadol could not be concluded as the pre-defined non-inferiority margin was exceeded.

**Conclusions:**

With both concepts, mean maximum pain intensity during exercise within the first 24 h after orthopedic/trauma surgery was reduced to a score of <4. This analgesic efficacy came at the cost of mainly gastro-intestinal side effects. Thus, we now use a prophylaxis against nausea and vomiting and pre-emptive laxatives as part of these concepts.

**Trial registration:**

https://eudract.ema.europa.eu (EudraCT- Nr. 2011–003238-15); October 24th, 2012.

## Background

A large prospective cohort study comparing the painfulness of 179 surgical procedures revealed that even ‘minor’ operative procedures may be associated with considerable pain (mean maximum pain scores within the first 24 h of 6–7 on the numeric rating scale (NRS) to 10) [1]. The 40 procedures associated with the highest mean pain scores included 22 distinct orthopedic/ trauma surgery procedures [1]. So why was insufficient analgesia so common? Most participating hospitals routinely used multimodal pain concepts based on oral opioids in combination with non-opioids, but the study patients had received rather low cumulative oral opioid doses compared to studies reporting on postoperative patient-controlled i.v. (i.v. PCA) morphine administration [1]. Moreover, pain scores in musculoskeletal surgery patients may not decrease at all during the first 24 h after surgery [2]. Thus, optimization of peri-operative analgesia regimens with respect to meeting procedure-specific opioid analgesic requirements on the one hand and minimizing opioid-related side effects on the other hand still constitutes an urgent medical need and a major clinical challenge.

Previous studies have shown that preemptive analgesia regimens based on oral extended-release opioids (oxycodone or buprenorphine, respectively) -starting with the first dose as premedication- provided improved pain control with a lower rate of side effects compared to conventional i.v. morphine PCA in spine and orthopedic surgery [3–6]. However, when trauma surgery patients were treated with oxycodone, the rate of constipation was high (46%) [7]. One way of addressing this particular side effect is an oral post-operative pain regimen based on the fixed combination of oxycodone with the μ-opioid receptor antagonist naloxone. Oral naloxone has a low bioavailability (< 3%) and, therefore, is supposed to exert its opiate antagonistic effects almost entirely in the gut to ameliorate opiate-induced bowel paralysis. However, the risk of nausea and vomiting was unaffected by naloxone [8, 9]. As an alternative, tapentadol, an opioid with a dual mode of action (μ-receptor agonist/ norepinephrine reuptake inhibitor) is now available for the treatment of medium to severe pain conditions. Efficacy/safety of tapentadol immediate-release (IR) in post-operative pain management have been documented [10–12]. In both acute and chronic pain, tapentadol was associated with less gastro-intestinal side effects compared to oxycodone [13, 14]. The aim of this study was to evaluate and compare an analgesic regimen based on extended-release (ER) oxycodone/naloxone (Targin®, Mundipharma GmbH, Germany) to a regimen based on extended-release tapentadol (Palexia®, Grünenthal GmbH, Germany) in trauma surgery patients with respect to (1) analgesic efficacy and (2) safety as revealed by the incidence of opioid-induced side effects (nausea, vomiting, constipation, sedation, vertigo and somnolence, respectively). The study was powered to detect superiority of tapentadol for at least one endpoint pending statistical proof of non-inferiority of tapentadol for both endpoints in a first step.

The study design was based on equianalgesic starting doses – either 10 mg oxycodone or 50 mg tapentadol [10, 11, 15–17].

## Methods

### Study design and study population

This randomized controlled observer-blind prospective clinical trial was approved by the German Competent Authority (BfArM, Nr. 4,038,588) and the Ethics Committee of Hannover Medical School (Nr 5897 M), and was registered at https://eudract.ema.europa.eu (EudraCT number 2011–003238-15) by Gertrud Haeseler October 2012. Principal investigator was Dirk Schaefers. The study was conducted at two hospital sites (Marien-Hospital Marl and St. Elisabeth-Krankenhaus Dorsten, later referred to as study sites 1 and 2) operated by one trust (Katholisches Klinikum Ruhrgebiet Nord GmbH, Marl, Germany) in cooperation with scientists affiliated at Hannover Medical School. Adult (≥ 18 years) trauma surgery patients ASA I-III were enrolled after written informed consent. Exclusion criteria were: inability to give informed consent, pregnancy or breast-feeding, pre-existing chronic pain treated with opiates, severe renal or hepatic impairment, and treatment with MAO- inhibitors.

### Study procedures and peri-operative management

Patients were randomized to receive either the test intervention tapentadol ER (Palexia retard®) or the control intervention oxycodone/naloxone ER (Targin®) by the Institute for Biostatistics, Hannover Medical School. Randomization was stratified for sex and study site.

The first dose of tapentadol (50 mg) or oxycodone/naloxone (10/5 mg) was given as premedication. The analgesic regimen then continued according to the initial allocation. Anesthesia was induced and maintained with propofol 4 mg/kg/h and remifentanil 0.15–0.25 μg/kg/min as total intravenous anesthesia (TIVA) adjusted to obtain stable cardiovascular parameters with no fluctuations in response to surgical stimuli. Adjunctive long-acting intravenous opioids and PONV prophylaxis were at the discretion of the anesthetist and subject to secondary analysis. Risk factors for post-operative nausea and vomiting (PONV) like female sex, no smoking habit, anticipated post-operative opioid analgesia and history of PONV were documented.

Post-operative pain in the recovery room was treated with intravenous piritramide and the second oral dose of study medication. The patient was observed for an hour following the second dose of study medication and discharged when he was comfortable without further opioid requirement. The cumulative dose of oral study medication (tapentadol or oxycodone/naloxone, respectively) established as effective (premedication dose plus eventual supplemental doses in the recovery room) was continued 2× daily on the surgical ward. Analgesia was supplemented with non-opioids dipyrone or acetaminophen, and/or a coxib, respectively, to achieve a residual pain score up to 3–4. In case that this level of analgesia was not obtainable, a trauma surgery consultant was involved to address surgical reasons. If there were none, opioid doses were increased. The doses of coxibes were not standardized in this study as we included ASA I-III patients and had no restrictions with respect to higher age, cardio-vascular disease and /or moderately comprised renal function- which may represent contra-indications for the use of coxibes. Thus, coxibes were prescribed after individual risk/benefit-analysis.

#### Rescue-interventions

- Nausea and vomiting: metoclopramide, ondansetron, dimenhydrinate and/or droperidol
- Constipation: As gastro-intestinal tolerability with respect to constipation is reported to be higher in both tapentadol and oxycodone/naloxone compared to oxycodone without naloxone, routine prophylactic laxatives are not generally recommended and we used the following rescue medication when constipation was observed: oral PEG 3350 (macrogol); oral sodium picosulfate, oral or rectal bisacodyl, oral lactulose
- Central-nervous side effects (sedation, vertigo, somnolence): Reduction of study medication

**Fig. 1:**
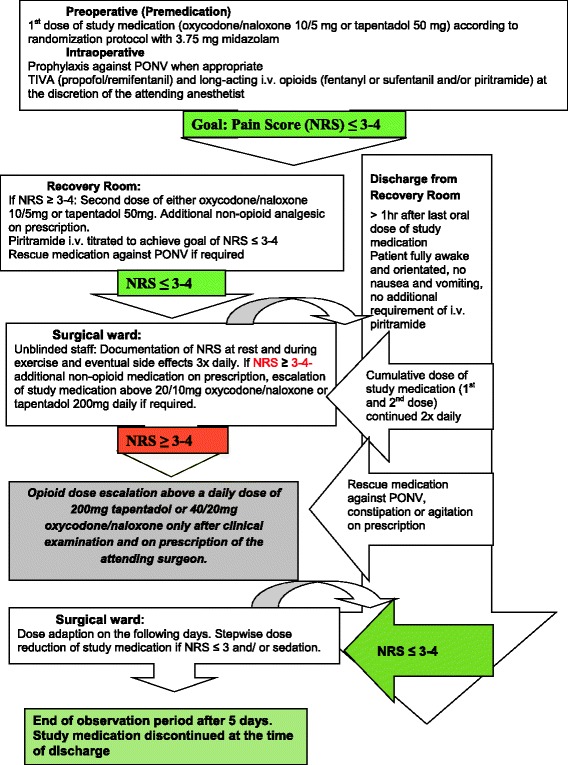
Algorithm of analgesic therapy and concomitant medication

When pain intensities decreased, the study medication was stepwise reduced. The treatment algorithm is depicted in Fig. 1.

### Data collection

The maximum pain levels quantified by patients on a numeric rating scale (NRS) from 0 (no pain) to 10 (maximum conceivable pain) during exercise and at rest as well as opioid side effects incidence and severity were obtained once a day by a blinded investigator and 3-times daily by non-blinded staff. Constipation was noted when the patient had no daily defecation. Sedation included only mild levels of drowsiness with the patient easy to awake as opposed to somnolence where the patient was difficult to awake. All therapeutic interventions including dosing of the study medication, of additional non-opioid analgesics and rescue medication were documented by non-blinded investigators. Dosing of long-acting intravenous opioids during anesthesia and in the recovery room and PONV risk factors and prophylaxis were obtained from the anesthesia protocols. The primary endpoints were derived from blinded observations only.

### Primary endpoints

There were 2 co-primary endpoints:

1. Analgesic efficacy defined as the mean NRS level during exercise over the 5 days observation period.
2. Safety measured by the incidence and severity (score 1–2) of side-effects. Score 1 is side effect observed but no requirement for intervention, 2 is side effect requiring intervention. The sum scores for each side effect over the five days observation period were calculated (maximum: 10) as well as the overall sum score across all side effects (maximum: 60). We defined, that a patient had a relevant side effect event at a sum score over the 5-days observation period of

- constipation ≥1
- nausea ≥3
- vomiting ≥3
- somnolence ≥1
- sedation ≥3
- vertigo ≥3, and/or
- overall sum score ≥ 5.

The primary endpoint was occurrence/non-occurrence of relevant side effect events. The differential weighted ranking of the severity of the side effects took into account that constipation and somnolence are potentially dangerous, resulting in either bowel paralysis or respiratory depression. Nausea, vomiting, sedation and vertigo were considered clinically relevant only if they persisted at least during two days of opiate therapy with intervention, or during three days without intervention. The decision to intervene in case of side effects was individualized according to the patient’s need.

### Primary objectives

Superiority of the tapentadol-based regimen would only be concluded when tapentadol was superior in at least one of the co-primary endpoints (pain or side effects) and not inferior in any of the two co-primary endpoints. Thus, the study formally had four primary objectives

1. tapentadol is non-inferior to oxycodone/naloxone regarding pain during exercise with a non-inferiority margin of 1, and
2. tapentadol is non-inferior to oxycodone/naloxone regarding occurrence of relevant side effects with a non-inferiority margin of 5%, and
  either
3. tapentadol is superior to oxycodone/naloxone regarding pain during exercise
  or
4. tapentadol is superior to oxycodone/naloxone regarding occurrence of relevant side effects (or both (3) and (4)).

### Secondary endpoints and objectives

- Mean NRS-level at rest
- Separate analysis of patients with high pain scores at the end of the observation period (NRS ≥ 5)
- Required daily doses of study medication, concomitant non-opioid medication and intra-operative long-acting intravenous opioids
- Type and dosing of rescue medication
- Analysis of expected and unexpected adverse events
- Total duration of post-operative opioid requirement
- Patient satisfaction at the end of the 5 days observation period

### Sample size calculation and statistical analysis

Sample size estimation was performed in nQuery Advisor 7.0. It was predefined that the tapentadol-based regimen was superior if three of the four hypotheses were proven. The one-sided type-I-error was α = 2.5% for the non-inferiority hypotheses (1) and (2) and α = 1.25% for the superiority hypotheses (3) and (4) with a Bonferroni-correction. In order to reach an overall power of 80%, the power for each objective was set to 93%. A two-group t-test, and a two-group t-test for equivalence, respectively, were used for sample size calculation for the objectives related to pain. For the objectives related to the occurrence of side effects, a chi-square test with continuity correction and an equivalence test for the comparison of two proportions were used. Data were available from a phase III clinical study showing a more than 5% higher incidence of gastro-intestinal side effects with oxycodone compared to tapentadol [11], and from a pre-study observation showing higher pain scores (one point) in the oxycodone/naloxone group with a standard deviation of 1.4 and, at the same time, a 21% higher incidence of side effects (35% vs. 14%). The overall sample size of the study was resulting from the objective with the highest necessary sample size, i.e. *n* = 123 patients per group as calculated for hypothesis (4). A low drop-out rate (<5–10%) was expected due to the inpatient setting and the short duration of observation. The final sample size to be recruited was increased to *n* = 133 patients per treatment arm.

### Primary population and analysis

In the analysis of the non-inferiority hypotheses, the per-protocol (PP) population was used. Superiority hypotheses were evaluated in the intention-to-treat (ITT) population. For the analysis of pain during exercise, a linear regression model was applied with the mean pain level of the first 5 post-operative days as dependent variable, and treatment, center and sex as independent factors. The treatment difference in pain and the corresponding 95% confidence interval (CI) were derived from the regression analysis and compared to the predefined non-inferiority margin of 1. Evaluation of treatment differences in the occurrence (yes/no) of relevant side effects was done using a Mantel-Haenszel-estimator for the risk difference, stratified by center and sex. The corresponding 95% confidence interval was calculated and compared to the predefined non-inferiority margin of 5%.

### Secondary analyses

Demographic and baseline characteristics, dosing of concomitant analgesic and rescue medication were compared by a two-sample t-test for numeric variables. Dichotomous secondary outcome data like incidence of side effect events yes/no were compared using a chi-square test. Associations between treatment group and secondary continuous outcome variables (e.g. pain at rest, treatment duration, and patient satisfaction) were analysed by linear regression with study medication as independent factor adjusted for study site and sex. Effect sizes are presented as differences in means with corresponding 95% CIs. The primary analysis was adjusted for sex and study site. The influence of further risk factors, e.g. BMI as continuous variable or PONV prophylaxis, was analysed by extended regression models (including treatment, center and sex, and additional risk factors). Again, linear regression was used for continuous outcomes. Logistic regression was used for categorical outcomes, especially occurrence of side effects. Odds ratios with corresponding 95% CIs are presented.

## Results

**Fig. 2:**
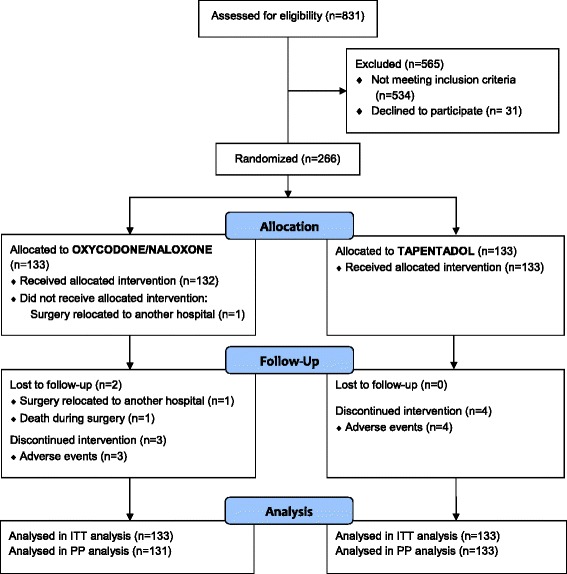
Study flow chart. A total of *N* = 266 patients were enrolled, 133 in each treatment arm. Two patients of the oxycodone/naloxone group and no patients in the tapentadol group were lost to follow-up. Reasons for loss to follow-up were not related to the allocated treatment (1× logistic reasons before pre-treatment and surgery, 1× death following surgery due to pulmonary embolism)

A total of 831 patients were assessed for eligibility. Of these, 266 patients were randomized to tapentadol (*n* = 133) and oxycodone/naloxone (*n* = 133) within a time frame of two years. Only two patients were lost to follow-up (1× fatal pulmonary embolism during surgery unrelated to the study, 1× transfer to another hospital before surgery), see Fig. 2.

Baseline patient characteristics were similar in the two study groups, see Table 1.

**Table 1:** Baseline characteristics of the per-protocol study population

Patient and surgery characteristics	Tapentadol (*n* = 133)	Oxycodone/Naloxone (*n* = 131)	Total (*n* = 264)
Study site			
1	65 (49%)	65 (50%)	130 (49%)
2	68 (51%)	66 (50%)	134 (51%)
Age (in years)	60 ± 19	59 ± 19	59 ± 19
Female sex	86 (65%)	83 (64%)	169 (64%)
Smoker	27 (20%)	26 (20%)	53 (20%)
BMI (in kg/m^2^)	27 ± 6	26 ± 5	27 ± 5
Number of risk factors for PONV			
1	16 (12%)	14 (11%)	30 (11%)
2	38 (29%)	43 (33%)	81 (31%)
3	61 (46%)	56 (43%)	117 (44%)
4	18 (14%)	18 (14%)	36 (14%)
Prophylaxis of PONV			
None	49 (37%)	56 (43%)	105 (40%)
Single	56 (42%)	50 (38%)	106 (40%)
Double	27 (20%)	25 (19%)	52 (20%)
Triple	1 (0.8%)	0 (0.0%)	1 (0.4%)
Type of surgery			
Open reduction and internal fixation (ORIF)	103 (77%)	98 (75%)	201 (76%)
Closed reposition and intramedullary nailing	10 (8%)	9 (7%)	19 (7%)
Joint replacement	9 (7%)	14 (11%)	23 (9%)
Reconstruction of ligaments and muscle	10 (8%)	10 (8%)	20 (8%)
Fixateur externe	1 (0.8%)	0 (0.0%)	1 (0.4%)
Duration of surgery (in minutes)	78 ± 41	78 ± 43	78 ± 42

### Primary efficacy results (pain reduction during exercise in the two study groups)

**Fig. 3:**
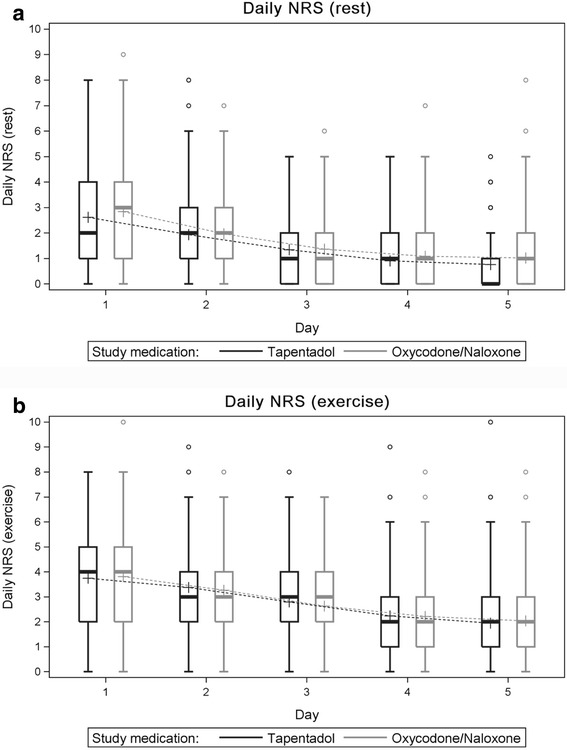
**a**, *top*) Pain scores at rest and **b**, *below*) pain scores during exercise during the first 5 days following the operation beginning with the day of the operation (day 1) on the NRS from 0 (no pain) to 10 (worst pain imaginable). *Box edges* indicate 25th and 75th percentiles. Whiskers indicate 5th and 95th percentiles. Pain intensities at rest were low already on day 1 in both groups, pain intensities during exercise decreased with time to a mean level of 2 in both groups on day 5

Mean adjusted daily pain levels during exercise in the first five post-operative days were 2.8 ± 1.3 (tapentadol, mean ± SD) and 2.8 ± 1.3 (oxycodone/naloxone; difference in means 0.03 [95% CI, −0.28 to 0.34]; *p* = 0.8). Mean maximum pain intensity during exercise in the first 24 h after surgery was 3.8 ± 1.9 (tapentadol) and 3.8 ± 2.1 (oxycodone/naloxone; difference in means −0.06 [95% CI, −0.54 to 0.42]; *p* = 0.8). Pain intensities during exercise decreased with time to a mean level of 2.0 ± 1.7 (tapentadol) versus 2.0 ± 1.5 (oxycodone/naloxone; difference in means −0.08 [95% CI, −0.46 to 0.30]; *p* = 0.7) on day 5. Maximum pain levels during exercise over five post-operative days beginning with the day of the operation (day 1) are depicted in Fig. 3b. In the primary analysis, non-inferiority of tapentadol regarding pain during exercise could be confirmed as the upper bound of the 95% confidence interval for the pain difference (tapentadol- oxycodone/naloxone) was below the pre-defined non-inferiority margin of 1. Superiority of tapentadol concerning pain during exercise could not be concluded.

There was a weak association between the cumulative dose (mg ME) of intra-operative i.v. opioids other than the short-acting remifentanil (fentanyl, sufentanil and/or piritramide) and pain reduction during exercise on day 1 (difference in means −0.02 [95% CI, −0.04 to 0.00]; *p* = 0.04). In the tapentadol group patients had received lower doses of long-acting opioids intra-operatively (15 ± 9 vs. 18 ± 13 mg morphine equivalent (*p* < 0.03)). There was no difference between the groups with respect to post-operative piritramide requirement and concomitant non-opioid analgesic medication (see Appendix 1 and Appendix 2).

### Primary safety results (differences between the study groups with respect to incidence of side effects)

Vomiting on day 1 occurred in 11% of the tapentadol and in 16% of the oxycodone/naloxone patients (*p* = 0.60). The incidence of constipation was 35% in the tapentadol vs. 30% in the oxycodone/naloxone group (*p* = 0.33). The incidence of a sedation/ vertigo event was <10%, somnolence <2% in both groups (*p* > 0.3, respectively). The incidence of side effect events in the two groups is depicted in Table 2. Despite similar scores of relevant side effects (tapentadol 51% vs. oxycodone/naloxone 49%; risk difference 3% [95% CI, −8 to 14%]; *p* = 0.6), non-inferiority of tapentadol could not be concluded because the upper bound of the 95% confidence interval for the risk difference (tapentadol – oxycodone/naloxone) was 14% and thus exceeding the predefined non-inferiority margin of 5%.

**Table 2:** Incidence of relevant side effect events

	Tapentadol	Oxycodone/naloxone	
	Frequency (Percent)	Frequency (Percent)	*p*-value (CHI^2^)
Nausea (≥3)	17 (13%)	23 (18%)	0.23
Vomiting (≥3)	4 (3%)	10 (8%)	0.09
Constipation (≥1)	47 (35%)	39 (30%)	0.33
Sedation (≥3)	8 (6%)	12 (9%)	0.33
Vertigo (≥3)	7 (5%)	5 (4%)	0.57
Somnolence (≥1)	3 (2%)	1 (0.8%)	0.32
Overall AE score (≥5)	40 (30%)	43 (33%)	0.63
Relevant adverse event	68 (51%)	63 (48%)	0.62

The severity of the respective side effect was rated with a daily score of 0–2, where 0 is absence of the respective side effect, 1 is side effect observed but no requirement for intervention, 2 is side effect requiring intervention. We defined, that a patient had a relevant side effect event at a sum score over the 5-days observation period of constipation and/or somnolence ≥1 and/or nausea, vomiting, sedation and/or vertigo ≥3, and/or an overall sum score ≥ 5

The unadjusted risk for nausea and vomiting was lower in the tapentadol group (Odds Ratios of 0.6 and 0.4), while the risk of constipation was higher (Odds Ratio 1.3). However, a multivariate regression analysis including PONV prophylaxis and risk factors to the logistic regression model revealed that the differences in the incidence of PONV between the treatment groups could be explained by confounding effects of PONV risk factors and PONV prophylaxis, now only leading to a treatment Odds Ratio of 0.98. Type and dose of rescue medication against nausea, vomiting and constipation is depicted in Appendix 3 and Appendix 4.

Incidence and severity of central-nervous side effects was low in both treatment arms. Somnolence was noted in three patients allocated to tapentadol and in one patient allocated to oxycodone/naloxone. In one patient (tapentadol) the finding of somnolence in the blinded observation was not consistent with the unblinded observation. In the three other cases, passing somnolence was noted in the immediate post-operative period in the intermediate-care unit in higher aged patients not requiring discontinuation of study medication.

### Secondary results: Pain at rest, dosing of post-operative study medication

Mean pain at rest in the first five post-operative days was 1.5 ± 1.1 (tapentadol, mean ± SD) versus 1.7 ± 1.2 (oxycodone/naloxone; difference in means −0.14 [95% CI, −0.42 to 0.13]; *p* = 0.3. Mean maximum pain at rest in the first 24 h after surgery was 2.6 ± 2.0 (tapentadol) and 2.8 ± 2.0 (oxycodone/naloxone; difference in means −0.23 [95% CI, −0.71 to 0.25]; *p* = 0.3) and decreased to 0.8 ± 1.0 (tapentadol) and 1.0 ± 1.4 (oxycodone/naloxone; difference in means −0.25 [95% CI, −0.04 to 0.54]; *p* = 0.1) on day 5. Maximum pain levels at rest over five post-operative days beginning with the day of the operation (day 1) are depicted in Fig. 3 A. Median pain at rest on day 1 was surprisingly low (<2) in the tapentadol group despite the fact that in the tapentadol group patients had received lower doses of long-acting opioids intra-operatively.

**Fig. 4:**
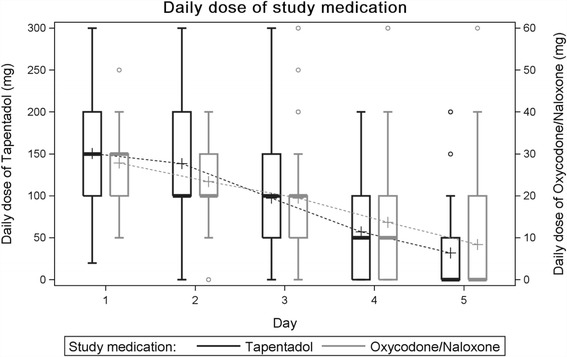
Post-operative daily dose of study medication (mg): Previous studies have shown that 50 mg of tapentadol is the equivalent dose to 10 mg of oxycodone. The following *boxplot* has two y-axes for the doses of each treatment arm. *Box edges* indicate 25th and 75th percentiles. Whiskers indicate 5th and 95th percentiles. Mean daily dose of study medication over the 5-days observation period did not differ between the groups

Mean equianalgesic daily dose of study medication over the 5-days observation period did not differ between the groups. The mean daily oxycodone equivalent dose was 19.0 ± 9.4 mg (tapentadol) versus 18.5 ± 9.4 mg (oxycodone/naloxone; difference in means 0.52 [95% CI, −1.73 to 2.77]; *p* = 0.6). Daily doses of post-operative study medication during the 5-days observation period are depicted in Fig. 4.

The cumulative doses of study medication for the different types of procedures are depicted in Table 3. Reconstructions of ligaments and muscle like rotator cuff repair or anterior cruciate ligament reconstruction were associated with the highest doses of study medication.

**Table 3:** Cumulative study medication during the 5-days observation period for the different types of procedures

Operation	Cumulative study medication (mg)	
	Tapentadol (mean ± std)	Oxycodone/naloxone (mean ± std)
Open reduction and internal fixation, ORIF	449 ± 228	94 ± 48
Closed reposition and intramedullary nailing	540 ± 238	82 ± 34
Joint replacement	567 ± 303	81 ± 43
Reconstruction of ligaments and muscle	580 ± 199	104 ± 64
Fixateur externe	700	.

### Secondary results: effects of sex and BMI, differences between the study sites

The effect of female sex on pain during exercise was not significant (difference in means 0.27 [95% CI, −0.058 to 0.59]; *p* = 0.1). Analyses additionally adjusted for BMI gave the same results like the primary analysis. Female sex was an independent risk factor for the occurrence of side effects (Odds Ratio 3.3 [95% CI 1.9 to 5.8]; *p* < 0.001). BMI had no significant influence on the incidence of side effects (i.e. no effect greater than 6% with 95% confidence;Odds Ratio 1.0 [95% CI 0.95 to 1.06]; *p* = 0.8).

Subgroup analysis of the primary hypotheses at the two study sites yielded the same results as the primary analysis. However, there were differences between the study sites which, apparently, did not affect the primary results: Mean (±SD) duration of the operation was 88 ± 44 min. at study site 1 and 68 ± 38 min. at study site 2 (*p* < 0.01) and only 60% of patients received intra-operative sufentanil or fentanyl at study site 1 (compared to 90% at study site 2). Mean daily pain intensities were significantly higher at study site 1 compared to study site 2: Mean pain during exercise was 3.1 ± 1.4 (study site 1, mean ± SD) vs. 2.5 ± 1.2 (study site 2; difference in means 0.61 [95% CI 0.30 to 0.92]; *p* < 0.001). Patients at study site 1 received higher mean daily doses of study medication for the first three days. Mean daily oxycodone equivalent dose over 5 days was 20.3 ± 9.1 mg (study site 1) vs. 17.3 ± 9.5 mg (study site 2; difference in means 3.0 [95% CI, 0.75 to 5.26]; *p* < 0.01). As a consequence, the incidence of relevant AEs was 62% at study site 1 compared to 38% at study site 2 (*p* < 0.001). Study site 1 was associated with a higher risk for side effects in the multivariate logistic regression model, probably as a result of higher doses of study medication (Odds Ratio 2.9 [95% CI 1.7 to 4.9]; *p* < 0.001).

### Separate analysis of patients with unacceptably higher pain scores at the end of the observation period (pain at rest ≥5 on days 4 or 5)

Higher pain scores at the end of the observation period in seven patients were in one case explained by inconsistencies with the non-blinded observation; in three cases study medication had been discontinued on day 2, in one case there was a surgical problem with an incorrectly placed splint. One patient (oxycodone/naloxone) had developed anxiety disorder with high pain scores unresponsive to opioid medication, in one patient (tapentadol) high pain scores were resulting from pre-existing spinal stenosis, not from pain at the operation site which was well controlled with low doses of study medication.

### Secondary results: treatment duration and patient satisfaction

**Fig. 5:**
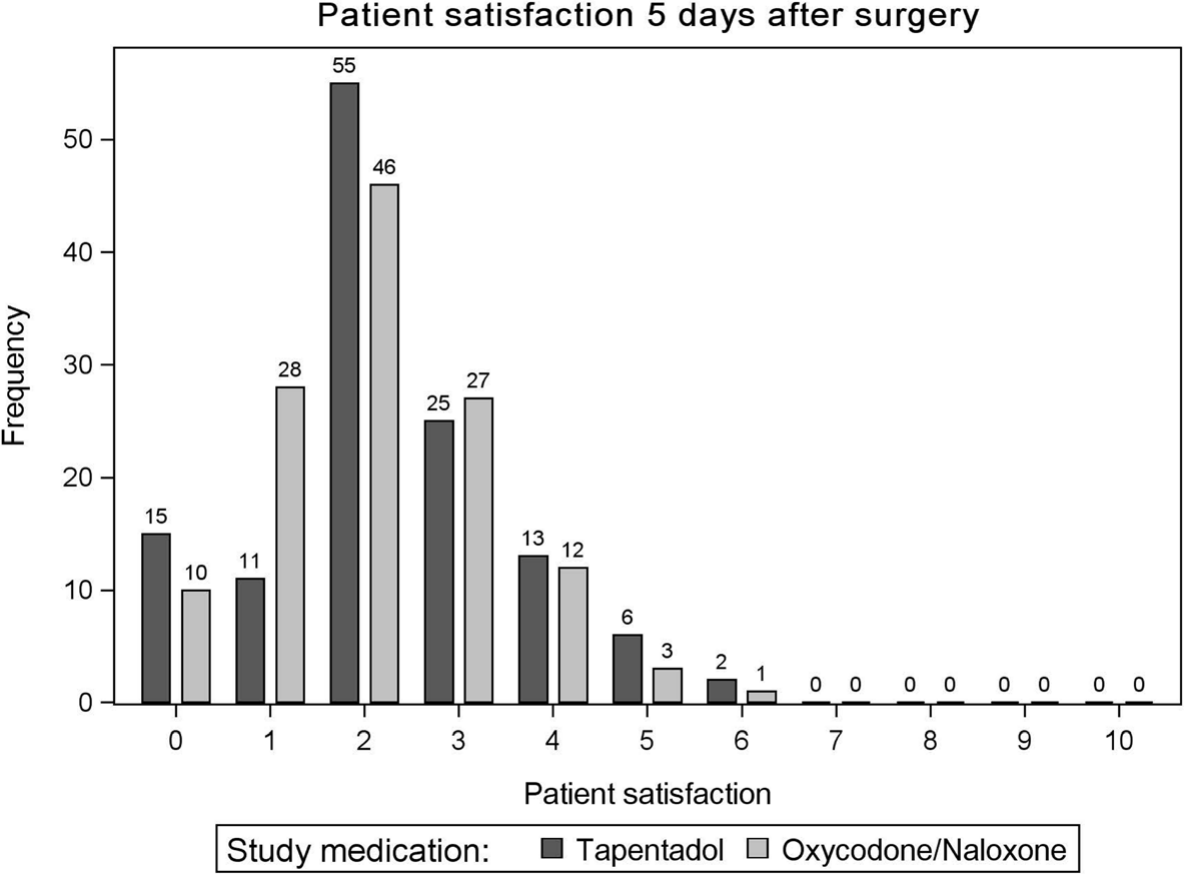
Patient satisfaction at the end of the study period from 0 (=very satisfied) to 10 (= not satisfied at all)

There were no differences regarding mean treatment duration with study medication (4.1 days in each treatment arm; mean difference 0.02 [95% CI, −0.49 to 0.46]; *p* = 0.95). Most patients were satisfied with their treatment. On a scale of 0 (very satisfied) to 10 (not satisfied at all) the mean score was 2.3 in the tapentadol group and 2.1 in the oxycodone/naloxone group (mean difference 0.14 [95% CI, −0.16 to 0.43]; *p* = 0.4). Patient satisfaction scores are depicted in Fig. 5.

### Secondary results: unexpected adverse events

The allover incidence of unexpected adverse events was comparable in the treatment arms (see Table 4). Only one SAE occurred unrelated to the study (oxycodone/naloxone group): A 90-year-old patient experienced a fatal pulmonary embolism confirmed by echocardiography following insertion of a femoral head prosthesis. Unexpected central nervous side effects probably related to the study medication were one case of optical hallucinations in the tapentadol group, one case of severe anxiety disorder and 4 cases of agitation and sleeplessness after discontinuation of study medication which we interpreted as withdrawl reactions in the oxycodone/naloxone group.

**Table 4:** Incidence of unexpected adverse events

Side effect	Tapentadol *N* = 133	Oxycodone/Naloxone N-133	Commentary
Central-nervous side effects			
Confusion	3 (0)		Confusion was observed in the immediate post-operative period, neurologic function recovered spontaneously while study medication was continued
Delirium		1 (1)	Delirium developed on treatment day 2 (20/10 mg daily dose), and resolved on day4 after discontinuation of study medication
Convulsion	1 (0)		Patient had a history of convulsions upon stress and sleep deprivation
Optical hallucinations (yellow circles)	1 (1)		Hallucinations disappeared two days after discontinuation of study medication
Mood disorders			
Euphoria, increased talkativeness	2 (2)		Euphoria resolved after dose reduction
Anxiety		1 (1)	Anxiety and catastrophizing reaction resolved when study medication was discontinued
Sleeplessness and agitation		4 (4)	Sleeplessness and agitation occurred following withdrawl of study medication
Cardio-vascular side effects			
Hypertension	1 (0)	1 (0)	Hypertension was noted pre-operatively
Dysrhythmia	1 (0)		Dysrhythmia was diagnosed pre-operatively
Hypotension	1 (1)		Blood pressure was restored to normal when study medication was discontinued
Other minor side effects			
Skin rash	1 (0)	1 (0)	
Sweating	3 (1)	5 (3)	4 cases attributed to metamizol
Dry mouth	3 (3)	2 (2)	(5) ameliorated after dose reduction
Aching stomach, heartburn	5 (1)	3 (1)	(2) ameliorated after dose reduction
Diarrhea	1 (0)	1 (0)	
Headache		1 (0)	

N = number of patients affected, in brackets number of cases potentially related to study medication

## Discussion

### Comparison of both analgesia concepts -analgesic efficacy and incidence of side effects

This study shows that a time-scheduled oral regimen with either extended-release tapentadol or oxycodone/naloxone in combination with non-opioid analgesics provided reliable pain relief following trauma/orthopedic surgery procedures- where pain management is still especially challenging. It is worth noting that mean NRS values for worst pain during exercise on the day of the operation (day 1) were <4 in our study in both groups- compared to 6–7 for trauma/orthopedic surgery in the large prospective cohort study presented by Gerbershagen et al. [1].

Our main hypothesis -non-inferiority of tapentadol compared to oxycodone/naloxone regarding pain during exercise- was confirmed, but neither non-inferiority of tapentadol with respect to side effect incidence nor superiority for analgesic efficacy and/ or side effect incidence could be established.

Still, the remarkably low median level of pain at rest on day 1 in the tapentadol group seen in our study merits further attention as it has been suggested that adrenergic descending inhibitory systems might be of potential relevance in (1) attenuating remifentanil-induced post-infusion hyperalgesia and anti-analgesia [18], and (2) in determining the time course of recovery from hypersensitivity after surgery [19].

The lower incidence of nausea and vomiting seen in tapentadol-treated patients could in part be explained by confounding factors of PONV (patients in the tapentadol group received more frequently a prophylaxis). Due to the differing use of PONV prophylaxis we could not show superiority of tapentadol with respect to PONV in this study. It has previously been established that the prevalence of nausea and vomiting in patients requiring opioids for post-operative analgesia (i.e. that have at least one risk factor) ranges between 21 and 79% [20]. The comparably high tolerability of both tapentadol and oxycodone/naloxone with respect to nausea and vomiting seen in our study may also be due to the extended-release formulation: The intermediate-release formulation of tapentadol was associated with an even higher incidence of nausea and vomiting (19% vomiting) when compared to oxycodone for acute post-operative pain relief after elective arthroscopic shoulder surgery [21]. In trials using intravenous patient-controlled analgesia 20–30% of patients presented with nausea and vomiting during the whole period of opiate requirement preventing patients from continuing opioid therapy [22]. The observation that the extended-release formulations apparently increase tolerability reaffirm the well-established concept that opioids are more dose-effective when stable plasma concentrations can be achieved over the whole duration of the painful stimulus [23].

Both oxycodone/naloxone and tapentadol were associated with a lower risk of constipation in trauma surgery patients compared to oxycodone without naloxone where the risk of constipation was 46% in trauma surgery patients [7].

However, despite higher gastro-intestinal tolerability of oxycodone/naloxone and tapentadol compared to i.v. opioids or oxycodone without naloxone, the problem of opioid-induced gastro-intestinal side effects in trauma surgery patients is not yet solved. Thus, based on our results, we recommend that established principles for the prophylaxis against PONV and opioid-induced constipation are pre-emptively applied in trauma surgery patients receiving opioids. Severe central nervous side effects like somnolence and/or confusion were rare (< 2%) and probably unrelated to study medications as neurologic function recovered spontaneously while study medication was continued. Only in one patient receiving 20/10 mg daily dose of oxycodone/naloxone study medication had to be discontinued due to delirium. No patient had opioid induced respiratory depression that required reversal with iv naloxone. Anxiety, sleeplessness and agitation was only seen in patients allocated to oxycodone/naloxone- in one patient anxiety resolved when oxycodone/naloxone was discontinued, in four cases agitation and sleeplessness occurred when oxycodone/naloxone had been withdrawn. These results indicate that psychomimetic effects of oxycodone and oxycodone withdrawal might constitute a problem even during short-term use.

The primary results on the effects of study medication were reproduced at two different study sites- despite the differences between the study sites with respect to duration of the operations and intra-operative opioid analgesic management. The fact that there was no association between sex, BMI and post-operative pain during exercise in trauma/orthopedic surgery is in accordance with a recent study showing no sex difference in post-operative pain in the joint surgery group [24].

### Non-responders

In six /264 patients no acceptable pain relief (NRS < 5) was provided by either treatment until day 4 or 5 of treatment duration. In three patients management has been inadequate (study medication discontinued at an early stage). In the three remaining patients, not responding to either tapentadol or oxycodone/naloxone was indicative of either a surgical problem or a wider, more complex medical issue (anxiety disorder/ neuropathic pain due to spinal stenosis) requiring particular attention by an experienced pain specialist. Thus, as previously suggested [25], it seems important to achieve effective analgesia in the early postoperative period and continue post-operative opioid treatment only as long as the nociceptive input from the wound persists after surgery. Patients that do not get pain-free during that time should be evaluated by a specialist in pain medicine and receive specific, preferably non-opioid treatment.

### Limitations of the study

The aim of our study was to evaluate and compare the efficacy and safety of both opioid-based analgesic regimens under the conditions of daily practice in our trauma surgery departments. As a consequence, there was a considerable heterogeneity in the types of surgeries performed, even within one category (i.e. open reduction and internal fixation- radial vs. femoral fracture) which might constitute a confounding factor. The postoperative exercise protocols differed with regard to the degree of mobilization and pain intensity. Still, it is interesting to note that while joint procedures are usually predicted to have the greater post-procedural pain, the amount of morphine equivalents consumed within that surgical category was similar to all other categories. Reconstructions of ligaments and muscle like rotator cuff repair or anterior cruciate ligament reconstruction were associated with the highest doses of study medication. However, the relatively small subgroup sizes limit the generalizability of these results. Another shortcoming of our study was the lack of patient blinding which might have biased the results.

## Conclusions

Both the tapentadol- as well as the oxycodone/naloxone-based perioperative multimodal oral analgesia concept provided reliable post-operative pain relief with mean pain scores over five days below 2 at rest and below 3 during exercise. The most common side effects were gastro-intestinal implying that trauma surgery patients receiving opioids might benefit from a routine prophylaxis against PONV and constipation. Analgesic failure with high pain levels at the end of the observation period despite continuing opioid medication was low (<2%) – and indicative of either a surgical problem or a complex pain condition independent from surgery. Severe anxiety and signs of withdrawl even after short-term use was only seen in oxycodone/naloxone- treated patients.

This study supports the existing clinical evidence in favor of the peri-operative use of oral extended-release opioids. Severe opioid-induced side effects like respiratory depression resulting in hypoxemic brain damage or death have been shown to be almost entirely associated with parenteral routes of opioid administration or combinations of different opioids [26]. For safety reasons, extended-release oral opioids should only be used in patients that have no restrictions for oral intake.

## Appendix 1

**Table 5:** Concomitant perioperative opioid analgesia in mg ME

	Tapentadol *N*=133	Oxycodone/Naloxone *N*=133	Total *N*=266
Dose of intra-OP fentanyl (mg ME)			
N	63	59	122
MEAN	13.4	15.7	14.5
STD	6.7	10.0	8.5
*p*-VALUE (T-TEST)			0.15
Dose of intra-OP sufentanil (mg ME)			
N	36	42	78
MEAN	15.8	21.9	19.1
STD	8.6	15.5	13.1
*p*-VALUE (T-TEST)			0.03
Dose of intra-OP piritramide (mg ME)			
N	67	52	119
MEAN	6.0	6.3	6.1
STD	2.7	2.6	2.7
*p*-VALUE (T-TEST)			0.6
Dose of intra-OP fentanyl or sufentanil and/or piritramide (mg ME)			
N	126	124	250
None	7	7	14
MEAN	14.4	17.5	15.9
STD	8.8	13.0	11.2
*p*-VALUE (T-TEST)			0.03
Dose of piritramide post-OP (mg ME)			
N	102	97	199
None	31	36	67
MEAN	8.0	7.0	8.0
STD	4.1	3.2	3.7
*p*-VALUE (T-TEST)			0.18

## Appendix 2

**Table 6:** Non-opioid analgesic medication

	Tapentadol		Oxycodone/Naloxone			
	N	Mean	N	Mean	*p*-value (Chi^2^)	*p*-value (t-test)
Acetaminophen (mg)						
Day 1	7	2214	4	1875		
Day 2	8	3313	6	3333		
Day 3	11	2727	6	3166		
Day 4	10	2750	6	3667		
Day 5	7	3286	7	3429		
Total over 5 days	16	7656	9	10278	0.14	0.32
Dipyrone (mg)						
Day 1	127	3484	124	3598		
Day 2	118	3353	114	3338		
Day 3	107	3339	105	3300		
Day 4	99	3247	95	3276		
Day 5	75	3127	80	3163		
Total over 5 days	130	13473	127	13605	0.31	0.85
Ibuprofen (mg)						
Day 1	44	873	38	953		
Day 2	68	1515	66	1424		
Day 3	68	1526	64	1469		
Day 4	70	1526	60	1440		
Day 5	67	1460	59	1403		
Total over 5 days	76	5918	67	5872	0.27	0.89
Etoricoxib (mg)						
Day 1	3	100	5	84		
Day 2	3	90	5	84		
Day 3	2	90	5	84		
Day 4	3	90	4	83		
Day 5	3	90	4	83		
Total over 5 days	6	215	6	320	1.00	0.24
Celecoxib (mg)						
Day 1	0	0	1	200		
Day 2	0	0	0	0		
Day 3	0	0	1	200		
Day 4	0	0	1	200		
Day 5	0	0	1	200		
Total over 5 days	0	0	2	400	0.16	-
Parecoxib (mg)						
Day 1	13	40	13	40		
Day 2	0	0	0	0		
Day 3	0	0	0	0		
Day 4	0	0	0	0		
Day 5	0	0	0	0		
Total over 5 days	13	40	13	40	1.00	1.00

Number of patients with the respective cumulative non-opioid pain medication in mg at day x and/ or more than 1 day “Total over 5 days”

## Appendix 3

**Table 7:** Rescue medication against nausea and vomiting

	Tapentadol		Oxycodone/Naloxone			
	N	Mean dose	N	Mean dose	*p*-value (Chi^2^)	*p*-value (t-test)
Metoclopramide (mg)						
Day 1	9	11	9	11		
Day 2	6	11	8	8		
Day 3	4	11	5	10		
Day 4	3	13	2	10		
Day 5	3	12	1	20		
Total over 5 days	15	19	21	12	0.28	0.24
Ondansetron (mg)						
Day 1	8	7	16	6		
Day 2	3	7	6	5		
Day 3	4	6	2	10		
Day 4	1	12	1	8		
Day 5	2	4	0	0		
Total over 5 days	12	10	19	8	0.18	0.52
Dimenhydrinate (mg)						
Day 1	1	62	1	62		
Day 2	0	0	0	0		
Day 3	0	0	0	0		
Day 4	0	0	0	0		
Day 5	0	0	0	0		
Total over 5 days	1	62	1	62	1.00	-
Droperidol (mg)						
Day 1	1	1.25	1	3.75		
Total over 5 days	1	1.25	1	3.75	0.99	-

Number of patients with the respective rescue medication in mg at day x and/ or more than 1 day “Total over 5 days”

## Appendix 4

**Table 8:** Rescue medication against constipation

	Tapentadol		Oxycodone/ Naloxone			
	N	Mean dose	N	Mean dose	*p*-value (Chi^2^)	*p*-value (t-test)
PEG3350 (g)						
Day 1	0	0	1	7		
Day 2	6	28	3	24		
Day 3	12	27	10	22		
Day 4	12	28	8	25		
Day 5	10	32	9	24		
Total over 5 days	17	68	13	55	0.44	0.47
Sodium picosulfate (mg)						
Day 1	0	0	0	0		
Day 2	1	10	0	0		
Day 3	3	10	3	6		
Day 4	6	9	2	10		
Day 5	2	8	0	0		
Total over 5 days	10	11	5	7	0.18	0.07
Bisacodyl (mg)						
Day 1	1	5	0	0		
Day 2	0	0	0	0		
Day 3	0	0	0	0		
Day 4	2	10	1	5		
Day 5	1	20	0	0		
Total over 5 days	4	11	1	5	0.18	0.44
Lactulose (g)						
Day 1	0	0	0	0		
Day 2	0	0	1	7		
Day 3	0	0	1	7		
Day 4	2	10	1	7		
Day 5	2	13	1	7		
Total over 5 days	3	16	4	7	0.70	0.13

Number of patients with the respective rescue medication in mg at day x and/ or more than 1 day “Total over 5 days”

## Abbreviations

AE: Adverse event
ASA: Health status according to the American society of anesthesiology
CI: Confidence interval
CL: Confidence limit
ER: Extended release
IR: Intermediate release
ITT: Intention to treat
MAX: Maximum
ME: Morphine equivalent
MIN: Minimum
NRS: Numeric rating scale (from 0 = no pain to 10 = max. Pain)
PCA: Patient-controlled analgesia
PONV: Post-operative nausea and vomiting
PP: Per-protocol
SAE: Serious adverse event
SD: Standard deviation
